# Design and Implementation of a Video/Voice Process System for Recognizing Vehicle Parts Based on Artificial Intelligence

**DOI:** 10.3390/s20247339

**Published:** 2020-12-21

**Authors:** Kapyol Kim, Incheol Jeong, Jinsoo Cho

**Affiliations:** Gachon University, Seongnam 1342, Korea; kapyol@ctsoft.kr (K.K.); kaddo@gc.gachon.ac.kr (I.J.)

**Keywords:** YOLO V3, SSIM, MSSIM, object recognition, speech recognition

## Abstract

With the recent development of artificial intelligence along with information and communications infrastructure, a new paradigm of online services is being developed. Whereas in the past a service system could only exchange information of the service provider at the request of the user, information can now be provided by automatically analyzing a particular need, even without a direct user request. This also holds for online platforms of used-vehicle sales. In the past, consumers needed to inconveniently determine and classify the quality of information through static data provided by service and information providers. As a result, this service field has been harmful to consumers owing to such problems as false sales, fraud, and exaggerated advertising. Despite significant efforts of platform providers, there are limited human resources for censoring the vast amounts of data uploaded by sellers. Therefore, in this study, an algorithm called YOLOv3+MSSIM Type 2 for automatically censoring the data of used-vehicle sales on an online platform was developed. To this end, an artificial intelligence system that can automatically analyze an object in a vehicle video uploaded by a seller, and an artificial intelligence system that can filter the vehicle-specific terms and profanity from the seller’s video presentation, were also developed. As a result of evaluating the developed system, the average execution speed of the proposed YOLOv3+MSSIM Type 2 algorithm was 78.6 ms faster than that of the pure YOLOv3 algorithm, and the average frame rate per second was improved by 40.22 fps. In addition, the average GPU utilization rate was improved by 23.05%, proving the efficiency.

## 1. Introduction

With the recent and rapid advancements in deep learning and hardware performance, object detection, which can be used to obtain information by automatically finding and classifying objects in an image, has become available in the field of image processing [1,2,3,4,5,6]. Such advances in object recognition technology have been implemented in artificial intelligence services, such as with robots, autonomous vehicles, and autonomous drones, enabling such machines to determine the current situation and take action autonomously without human intervention. Functions such as face, ID card, and license plate recognition can also be implemented and used in a variety of application services.

The number of used-vehicle sales in Korea has exceeded that of the new vehicle sales since 1998 and has been steadily increasing. In addition, the number of used-vehicle sales in 2018 was 2,602,198, which is 1.44-times larger than the number of new vehicle sales of 1,813,051. In 2018, the total used-vehicle sales amounted to 10.2492 trillion, and the market has been rapidly changing from offline to online sales [7,8]. This is a global phenomenon and is not confined to Korea, and despite the quantitative increase and external growth of the used-vehicle market, there are continuing problems and complaints from consumers owing to an opacity of the distribution channels and information on the used vehicles being sold. In particular, inconsistencies in used-vehicle information are increasingly harming consumers owing to the nature of the non-face-to-face sales of online markets.

In this study, to convert the vehicle parts into objects and the vehicle description from the seller’s used-vehicle promotional video into text data, You Only Look Once (YOLO)v3 object recognition technology and Speech-to-Text (STT) were applied to a video-based online platform for used-vehicle sales. In addition, a system for determining whether a used vehicle is properly described in a video promotion was developed. A method is also proposed to increase the efficiency of the object recognition function by applying the mean structural similarity index (MSSIM), which measures the structural similarity of the original image to the YOLOv3 algorithm [9,10].

In this study, a system was developed that automatically filters user videos uploaded onto a used-vehicle sales platform through a video search, and reduces the number of computations by discriminating video frames in which changes to the object are insignificant. It is expected that the response speed and service experience of the proposed platform can be improved by increasing the object recognition speed without changing the network of the object recognition algorithm.

## 2. Related Research

### 2.1. YOLO Algorithm

Object recognition algorithms rapidly advanced after the convolutional neural network (CNN)-based AlexNet [11], developed by Krizhevsky, Hinton, and Sutskever, won the 2012 ImageNet Challenge, and reached recognition levels higher than those directly obtainable by human viewers at the 2015 ImageNet Challenge [12,13,14].

Since then, many researchers have investigated methods for classifying and recognizing complex objects in natural images and classified complex objects using an algorithm called a region-based CNN (R-CNN), which considers the region. [15]. R-CNN has brought about innovative developments in the field of object recognition, and the fast-RCNN [16], faster-RCNN [17], and YOLO [18] algorithms were developed (in that order), and have reached the level of real-time object recognition [19].

**Figure 1:**
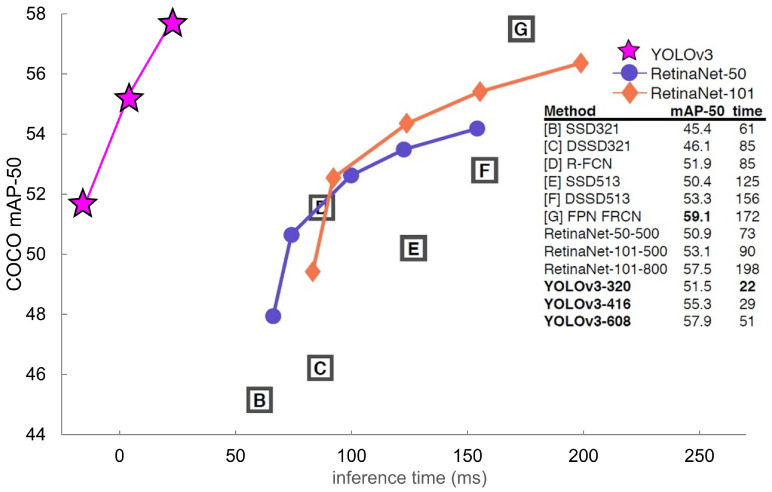
Performance comparison graph of artificial intelligence object recognition algorithms focused on YOLOv3 and RetinaNet analysis [21].

According to Figure 1, it takes 55.3 mAP (mean average precision) and 29 ms for YOLOv3 to analyze a single image based on the YOLOv3-416 model of an Nvidia GeForce GTX TITAN X graphics card used to train the COCO dataset (80 objects) [20], which is known to be complicated. These figures prove that, although the object recognition accuracy is similar to that of object recognition algorithms excluding YOLOv3, a better object recognition speed is achieved.

The YOLO algorithm divides an input image into S × S grid cells and predicts B bounding boxes and confidence scores inside each cell. The confidence score of the model indicates the probability that the predicted object is within the bounding box, and can be defined using the following equation:

(1) $$Pr\left(Object\right)\times IOU\frac{truth}{pred}$$

The bounding box consists of a total of five elements, namely, *x, y, w, h*, and the confidence, where *x* and *y* are the center coordinates of the bounding box based on the boundary of the grid cell, and *w* and *h* are the width to height of the predicted object.

**Figure 2:**
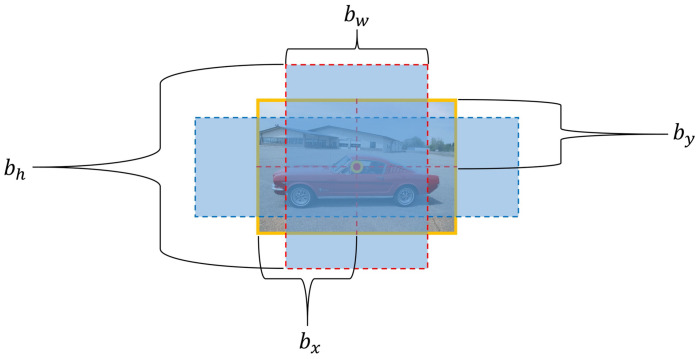
Structure diagram of bounding box according to $b_{x}$, $b_{y}$, $b_{w}$, and $b_{h}$ for YOLO image detection.

Each grid cell predicts C, the conditional class probability, as shown in Equation (2) below. This probability outputs only one type of class probability regardless of B, which is the number of bounding boxes per grid cell. The structure of the YOLO bounding box is as shown in Figure 2.

(2) $$Pr(Class_{i}|Object)$$

Finally, a class-specific confidence score can be obtained by multiplying the confidence score and conditional class probability as shown in Equation (3) below, which is a value that indicates the probability of a specific class object being found in the corresponding bounding box and whether the bounding box was correctly predicted for the object.

(3) $$Pr\left(Class_{i}|Object\right)\times Pr\left(Object\right)\times IOU\frac{truth}{pred}=Pr\left(Class_{i}\right)\times IOU\frac{truth}{pred}$$

**Figure 3:**
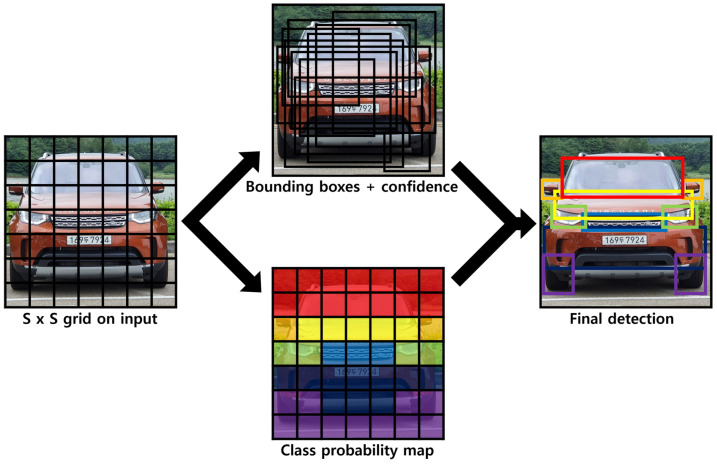
The object recognition process for images using YOLO grid analysis.

For example, if grid S = 7, bounding box B = 2, and C = 20, then a 7 × 7 × (2 × 5 + 20) output tensor can be obtained, where 5 indicates the five elements of the bounding box, and 20 is the conditional class probability value for the class. A class-specific confidence score of 7 × 7 × 2 can be obtained through the output tensor, and a non-maximum suppression is applied based on each of the 20 classes for 98 class-specific confidence scores; the class and bounding box for the object are then determined. This series of processes is illustrated in Figure 3.

**Figure 4:**
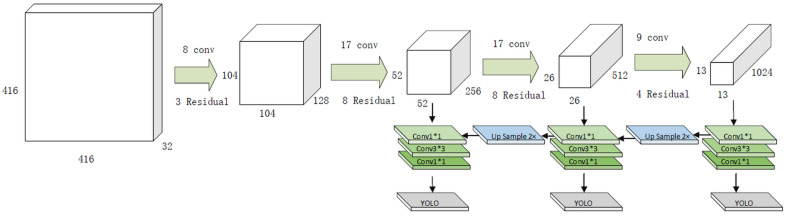
YOLOv3 network architecture.

The region proposal network (RPN) method used in faster-RCNN was adopted in the YOLOv2 and YOLOv3 algorithms. There is no need to infer the bounding box coordinates because the anchor box can be selected in advance. Therefore, a fully connected layer (FC Layer) is not used but a convolution layer is. In addition, although a class-specific confidence score of 7 × 7 × 2 = 98 was used in YOLOv1, five anchor boxes were substituted, and a class-specific confidence score of 13 × 13 × 5 = 845 was used in the YOLOv2 and YOLOv3 algorithms, thereby improving the performance compared to the conventional YOLOv1. This series of processes is illustrated in Figure 4.

The motives for selecting YOLOv3 in this study are as follows [21]:

- A fast speed with a simple processing is achieved.
- The value of mAP is approximately twice that of other conventional real-time detection systems.
- YOLOv3 is expected to improve the service response speed of the platform with real-time object recognition.
- As conventional artificial intelligence object recognition algorithms were developed using Python, their source code may be vulnerable to security risks.
- As the source code was released based on C language, it can be secured through a compilation.
- As YOLOv3 was also implemented based on C language, it has high development accessibility and easy maintenance.
- As it is easy to improve performance and expand the object types through dataset training, it can adapt easily to changes in the environment of the development platform.

In this study, the YOLOv3 algorithm was used to recognize objects for each internal and external part of the vehicle in a used-vehicle promotional video. To this end, the Darknet Framework code [22] was modified, and to optimize the application in a service model, a progress module was developed to calculate the moment when each object was recognized, including the object information of the used-vehicle part, and to achieve an asynchronous execution.

### 2.2. Application Service Using YOLOv3

The YOLOv3 algorithm is mainly used to build image detection applications for image analysis in various fields including medicine, agriculture, and transportation [23,24]. It has also been used as a core technology in the agriculture field for the automated detection of apple growth. In addition, the MD-YOLO framework was developed for automated real-time vehicle license plate detection in the field of transportation [6,25]. For autonomous driving, a Lidar-based approach was developed as the vehicle detection system, and the YOLOv3 algorithm was used for improving the system [26,27]. Likewise, the Yolo algorithm has the advantage of real-time object recognition and is being used as a core technology for automated object recognition in artificial intelligence systems.

However, the algorithm should be able to stably analyze at a rate of more than 60 fps, which is used in most video analysis, and the ability to analyze at higher than 100 fps is required for high-definition UHDTV video. In this study, the YOLOv3 algorithm was used to recognize objects for each internal/external part of the vehicle in a used-vehicle promotional video. As the promotional video supports a rate of 60 fps by default, the YOLOv3 algorithm needs to be improved. The average frame rate per second of major AI object recognition algorithms is shown in Table 1 below.

### 2.3. MSSIM Algorithm

**Figure 5:**
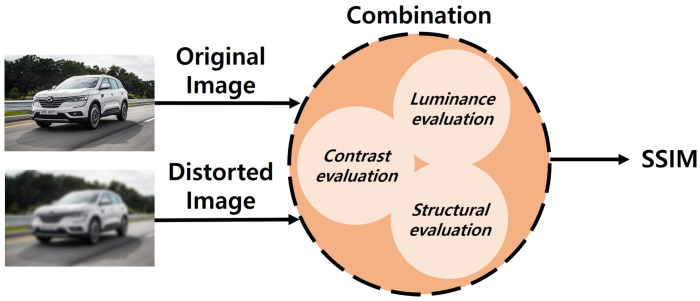
Schematic diagram of the image similarity evaluation of the SSIM algorithm.

The structural similarity (SSIM) algorithm was designed for evaluating the image quality and the differences and similarities in quality based on human visual perception, and is not a method for calculating numerical errors or conducting comparisons with the original image [28,29,30,31]. It is specialized in deriving structural information of images similar to the human visual system, and compares the luminance, contrast, and structure of the original image with those of a distorted image. A conceptual diagram of SSIM can be found in Figure 5.

Using the SSIM, the luminance and contrast ratio can be obtained through Equations (4) and (5):

(4) $$l\left(x,y\right)=\frac{2\mu_{x}\mu_{y}+C_{1}}{\mu_{x}^{2}+\mu_{y}^{2}+C_{1}}$$

(5) $$c\left(x,y\right)=\frac{2\sigma_{x}\sigma_{y}+C_{2}}{\sigma_{x}^{2}+\sigma_{y}^{2}+C_{2}}$$

With the SSIM, the structure is defined by subtracting the average luminance from the image and dividing it by the standard deviation, as shown in Equation (6).

(6) $$\frac{x-\mu_{x}}{\sigma_{x}},\frac{y-\mu_{y}}{\sigma_{y}}$$

The structures of the original image and a distorted image can be compared, as shown in Equation (7), by obtaining the correlation coefficients of *x* and *y*, which is equivalent to obtaining the correlation coefficient between the Equation (6) above and the correlation coefficients of *x* and *y*.

(7) $$s\left(x,y\right)=\frac{\sigma_{xy}+C_{3}}{\sigma_{x}\sigma_{y}+C_{3}}$$

In Equations (4)–(7), $C1={\left(0.01\times L\right)}^{2}$, $C2={\left(0.03\times L\right)}^{2}$, C3=C2/2, and L is the dynamic range of the pixel values. Finally, the SSIM can be obtained by defining the image quality map by combining Equations (4)–(7), which can be defined through the following:

(8) $$SSIM\left(x,y\right)=\frac{\left(2\mu_{x}\mu_{y}+c_{1}\right)\left(2\sigma_{xy}+c_{2}\right)}{\left(\mu_{x}^{2}+\mu_{y}^{2}+c_{1}\right)\left(\sigma_{x}^{2}+\sigma_{y}^{2}+c_{2}\right)}$$

(9) SSIM(x,y)=l(x,y)c(x,y)s(x,y)

The MSSIM is the average value of the SSIM obtained by grouping a 11 pixel × 11 pixel sized area, which can be calculated through Equation (10):

(10) $$MSSIM\left(X,Y\right)=\frac{1}{M}\sum_{j=1}^{M}SSIM\left(x_{j},y_{j}\right)$$

In this study, the MSSIM between video frames was obtained to compare the images, and was designed to determine whether the image object information obtained using the existing YOLOv3 remains unchanged when the structural change of the image is small. A GPU processor was used for a fast computations of the MSSIM.

## 3. Proposed System Design

### 3.1. Video Process System Design

The system proposed in this study can be mainly divided into two systems: video and voice processing systems. The video processing system is an artificial intelligence system that uses the YOLOv3 and SSIM algorithms to recognize vehicle objects from used-vehicle sales videos uploaded by users and output the results in Json format. The requirements of the view processing to be implemented are defined in Table 2 below.

**Figure 6:**
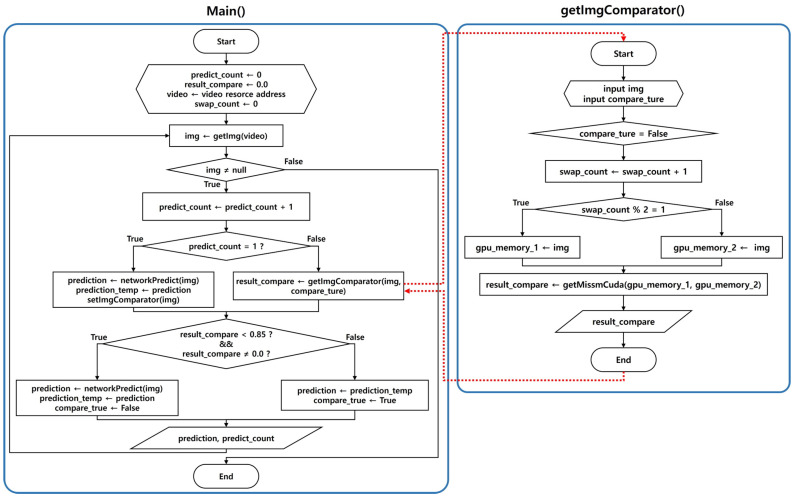
Operation algorithm flow of a video processing system including the MSSIM algorithm.

The key to the video processing system proposed in this study is to compare the similarity of the image of the previous frame with the currently input image based on the analysis results of the image extracted from the image uploaded by the user through the MSSIM image quality comparison algorithm. The system was designed to determine whether to newly analyze the current image through the YOLOv3 network function or apply the data analyzed in the previous frame according to the set comparison value. The operation process for the designed vehicle part recognition module operation algorithm is shown in Figure 6.

### 3.2. Voice Process System Design

The voice processing system is an artificial intelligence system that extracts the seller’s verbal description in a used-vehicle sales video using STT API, converts it into text, filters out any profanity and vehicle-related words from the converted text, and outputs the results in JSON format [32,33,34]. The requirements of the voice processing system to be implemented are defined in Table 3.

**Figure 7:**
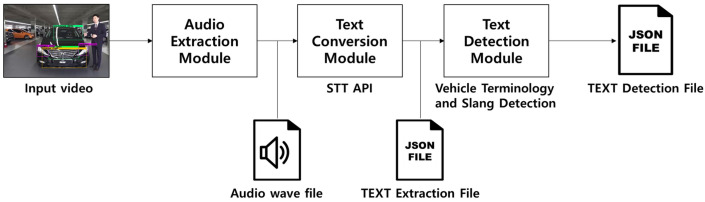
Voice process system operation algorithm flow.

The proposed voice processing system utilizes the STT API to extract the recorded voice from the used-vehicle promotional video uploaded by the seller and convert it into text every 59 s. It then determines whether the seller was describing the current vehicle and whether inappropriate language was used. It was designed to provide a service for limiting the exposure of images that were rejected by the system to customers using the filtering algorithms developed in this study and to provide a service allowing an editing request to be given to image uploaders. The operation process for the designed speech extraction and filtering algorithm is shown in Figure 7.

### 3.3. Video and Voice Processing System Administrator Service System Design

**Figure 8:**
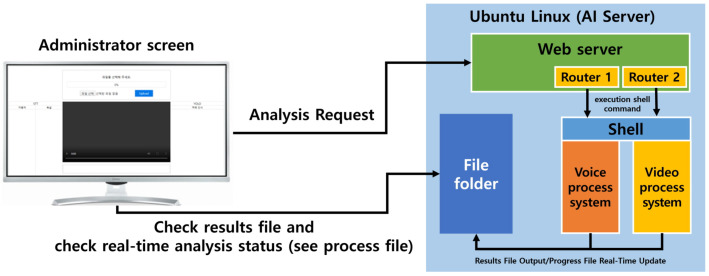
Configuration diagram of video and voice processing system administrator service system module.

The video process system and voice process system implemented in this study are provided on a video-based online platform for used-vehicle sales, and the administrator page of the platform is serviced through a web page. Therefore, an administrator service system was modularized and linked to service the video process system and voice process system on a web page. The basic layout of the administrator page was designed, as shown in Figure 8.

In the Figure 8 above, each system has a developed calling module based on the concept of a service router that can be called within a web server and return the result in the form of a file allowing each system to be operated in a web service environment, and the router executes each system asynchronously through a shell script. The video and voice processing system executed asynchronously returns the real-time progress and results through a file input/output, and the progress can be monitored in real-time using a polling method. The final result is then received on the administrator page.

## 4. Implementation of the Proposed System

### 4.1. Implementation of the Video Processing System

#### 4.1.1. Data Training for Vehicle Part Recognition

The video process system proposed in this study recognizes the vehicle parts based on YOLOv3 and was used to secure 23,794 vehicle images including passenger cars and SUVs [35]. To build a dataset through labeling, the vehicle parts displayed in each image were divided into 20 objects, and the ratio of training, validation, and testing was set to 7:2:1, and training was conducted through the YOLOv3 model [36,37]. The object recognition items are shown in Table 4.

**Figure 9:**
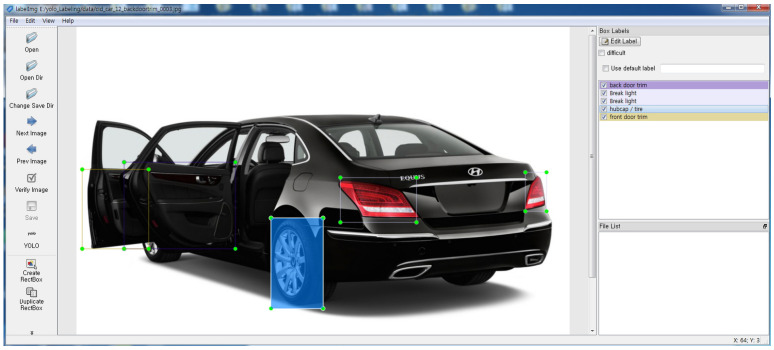
Labeling during training of vehicle parts using LabelImg program.

For convenience, the LabelImg program was used for the labeling of the training dataset, which was mainly composed of images with different exterior vehicle colors for cases in which the vehicle images have a similar composition [38]. Figure 9 shows the work of obtaining datasets using the LabelImg program.

#### 4.1.2. Vehicle Part Recognition Results of Transmission Module Development

For the video process system proposed in this study, when a used-vehicle seller uploads a video, the video is automatically analyzed based on the event of the administrator, and the recognition result of the vehicle part is saved and used as a JSON file. To this end, a DEMO.C file of DARKNET, the existing YOLOv3 framework, was modified and developed to meet the system requirements.

Output file settings and development:

- It was developed to provide a ResetFul API by structuring three types of output files for both vehicle and vehicle part recognition.
- File name_yolo.json: The user can receive the result through a JSON file containing information such as the recognized vehicle parts, location, and time.
- File name_yolo.mp4: A file copy displaying the vehicle parts recognized in the original video file was developed to visually check the vehicle parts recognized in the video file.
- File name_yolo.progress: As this system is executed asynchronously in real time in response to web service requests, the user should be able to check the progress. To this end, it is developed to continuously update the file that displays the current progress, and check messages regarding errors and exceptions.

The vehicle part recognition result is transmitted to the web service administrator through “file name_yolo.json”, as described above, the JSON file format of which is as shown in the following figure. The “frame_id” refers to the current frame number, “time” refers to the video playback time of the current frame, and “objects” refers to the object information of the vehicle parts recognized as an array. The format of the recognized vehicle part object was defined to also display the location and size in the video screen, and the accuracy of the recognized object, as shown in the following Table 5.

As the time value of the analyzed video cannot be checked in the existing DARKNET DEMO.C file shown in Table 5 above, an additional function was developed to calculate the time value by obtaining the frame rate per second and the count of the current frame. However, an error was found in which the time value of the analyzed frame and the original video frame were found to be out of sync, the cause of which was analyzed to fix the issue, as shown in the following Table 6.

#### 4.1.3. Proposal and Development for Improving Performance of Vehicle Part Recognition Module

**Figure 10:**
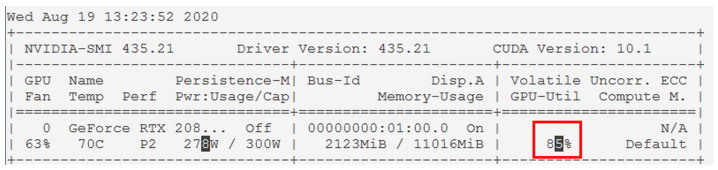
GPU utilization rate of YOLOv3 based on Nvidia GeForce RTX 2080Ti.

Although YOLOv3 is a good alternative that quickly and automatically recognizes objects in images by adopting the concept of real-time object detection, it requires a high-end computing environment, and is particularly sensitive to the GPU performance [21]. As shown in Figure 10, the GPU utilization rate was maintained at over 80% while analyzing the video using the YOLOv3 algorithm based on the Nvidia GeForce RTX 2080Ti graphics card. Owing to the characteristics of a web service with frequent service requests, there is concern regarding the service quality degradation because two or more service requests can only be processed sequentially.

In addition, the video uploaded to the used-vehicle sales platform using this system should be processed at a minimum rate of 60 fps for implementing a real-time service, which requires a rate of 24–60 fps. An analysis of a model trained to recognize 20 vehicle parts as objects using the YOLOv3 object recognition algorithm in the Nvidia GeForce RTX 2080Ti environment showed that a rate of 66 fps was maintained. Although the minimum requirements have been met, requirements for even a more efficient system are emerging.

In this study, in developing the video process system, the YOLOv3 object recognition algorithm and an MSSIM image quality comparison algorithm were used to increase the efficiency of the GPU utilization and obtain faster object recognition results. Rather than analyzing the entire 10 to 15 min video clip using the YOLOv3 algorithm, the vehicle parts were recognized as objects through the YOLOv3 algorithm under the appropriate conditions, and in some cases, a method to reduce the GPU utilization rate was applied by applying the object information of the vehicle parts recognized in the previous frame to the current frame. This idea is based on the characteristics of video frame information. Video clips are recorded at approximately 24–60 fps, and adjacent video frames have a remarkably small change in the number and location of the vehicle part objects unless there is a rapid structural change captured by the human eye. In other words, if a side-mirror object is recognized in frame 1, the side-mirror object of frame 1 is still recognizable in frame 2, and the location remains mostly unchanged.

**Figure 11:**
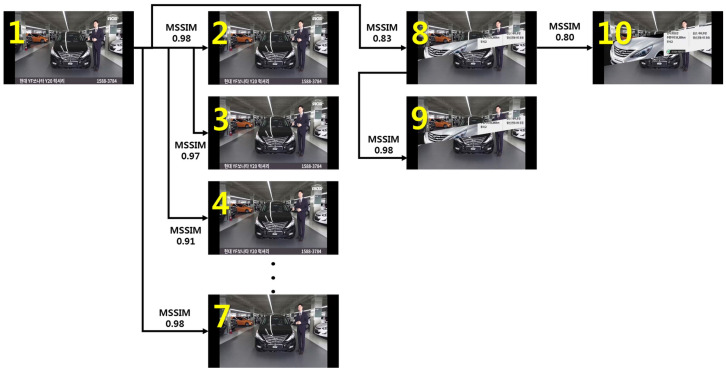
MSSIM results according to the change in video frame of the same vehicle.

As shown in Figure 11, for frames 2–7 based on frame 1, the result of the MSSIM image quality comparison algorithm is 0.9 or above, and the change in the visual image is insignificant. Therefore, the analyzed vehicle part object recognition information of frame 1 can be shared with frames 2–7. However, a clear difference is visible when comparing frames 1 and 8. As a result of an analysis using the MSSIM algorithm, the value was 0.83, and there was a structural change between the two images. In this case, the YOLOv3 algorithm can apply a procedure to find a new vehicle part object in frame 8. In other words, the reference frame and the frame to be compared are sequentially compared through the MSSIM algorithm, and the vehicle part object was newly recognized when the value of MSSIM was 8.5 or below.

**Figure 12:**
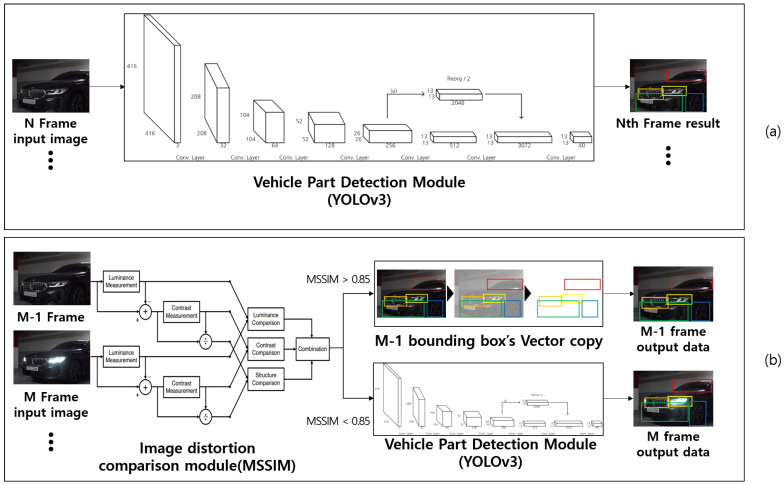
Structure diagram of vehicle part recognition module including the MSSIM algorithm: Operation structure of (**a**) the pure YOLOv3 algorithm and (**b**) the YOLOv3 algorithm including the MSSIM algorithm [41].

In general, the time required to compare the two images using the MSSIM algorithm is shorter and fewer computing resources are consumed compared with the analysis using the YOLOv3 algorithm. However, a new CUDA programming is required to program the MSSIM algorithm because the existing open CPU-based library or API cannot be operated on a GPU. In addition, image resources should be uploaded with caution to the GPU memory because doing so consumes a significant amount of computing resources and may affect the overall program operating time. Therefore, the programming was conducted under a condition in which image replacement using GPU memory was refrained as much as possible, as shown in Figure 12.

In general, object recognition artificial intelligence algorithms including the YOLOv3 algorithm shows the time complexity of O(n) owing to the iterative computations of the image arrays [39,40]. The vehicle parts recognition module proposed in this study had a time complexity of O(log n) because it does not analyze all images depending on the situation by using the MSSIM image processing technique. However, as a worst case, it can show the time complexity of O(n) as in conventional object recognition artificial intelligence algorithms.

The key point of Figure 12 above deals with the question of whether the image extracted from the video will pass the YOLOv3 network function according to the result of the MSSIM image quality comparison algorithm or whether the previous analysis will be applied.

In this study, when the MSSIM value of both images was below 8.5, the YOLOv3 network function was executed to update the new vehicle part object information. If the MSSIM value exceeds 8.5, not only are the structural changes insignificant to the naked eye, the YOLOv3 object recognition algorithm must also be executed frequently, which may increase the overall computing load.

**Figure 13:**
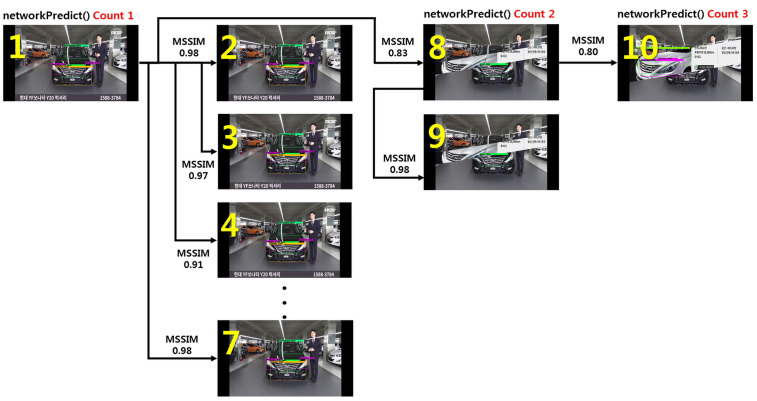
Object recognition according to changes in vehicle video frame through the vehicle part recognition module of the video processing system.

For the development of the video process system, this was modified, centering on the detect_in_thread() in the demo.c file of the Darknet framework, and the setImgComparator() and getImgComparator() functions were developed. In addition, CUDA programming was applied, allowing the MSSIM algorithm to be executed on the GPU, and then linked with detect_in_thread(). The result of executing the video process system based on the example in Figure 11 above is shown in Figure 13 below.

A total of four vehicle part objects were recognized in frame 1, and the structural change of the image from frames 2–7 was insignificant, and thus the information on four vehicle part objects was applied without any changes. A structural change of the image was found in frame 8, and thus one vehicle part object was newly recognized. The object information from frame 8 was applied to frame 9, and four vehicle part objects were newly recognized in frame 10. During the process of analyzing the 10 frames, the networkPredict() function was executed three times, in contrast to the networkPredict(), which was executed 10 times in the existing pure (original) Darknet Framework.

### 4.2. Voice Process System Implementation

#### 4.2.1. Voice File Extraction and Division Module Development

A voice process system is an artificial intelligence system that extracts the seller’s oral description in a used-vehicle sales video using the STT API, converts it into text, filters out any profanity and vehicle-related words from the converted text, and outputs the result in JSON format.

**Figure 14:**
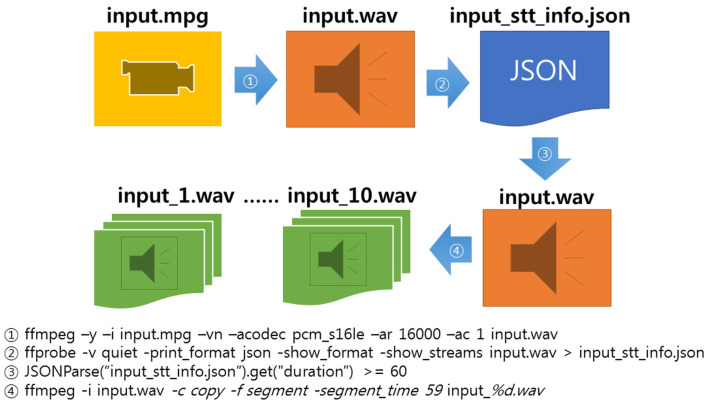
Audio file extraction and unit division process for extracting profanity and related words.

To use the STT API in the synchronization recognition mode used in this study, a module was developed that extracts an audio file from a video file uploaded by a user and divides the file in units of 59 s if the audio file is longer than 1 min. To extract audio files from a video, and acquire and divide the information, the ffmpeg program for Linux was implemented through Java, and operates as shown in Figure 14 below.

#### 4.2.2. TEXT Conversion Module Development

The text conversion module of the voice process system was developed by utilizing the synchronization recognition module of the STT API. The audio files divided through JAVA programming were managed through a fileList called ArratList, and syncRecognizeWords() was called according to the number of files. As it is a synchronization recognition module, it has the advantage of immediately converting audio files remotely into text but enters a standby state while all voice files are converted into text. Therefore, RsultThread() was implemented to obtain the standby time while each audio file was being analyzed, and “{fliseName}_stt.progress” file was created to notify the current progress to the web service, and update the contents until the current progress was completed.

According to Table 7, totalTime is the total playback time of the integrated audio file, and syncRecognizeWords() is a function that calls the STT API in synchronous recognition mode. While using the STT API, an asynchronous thread was created to continuously update the current progress through the file output, and the recognized TEXT was saved and managed in the jWordsArray variable in JSON format.

In the TEXT conversion module, text is detected in the audio file, and each text is grouped into word units and stored in a JSON array. The information can be searched and updated after converting the audio file into text, and the JSON array is defined as shown in Table 7 below.

#### 4.2.3. Vehicle Terminology and Profanity Extraction Module Development

**Figure 15:**
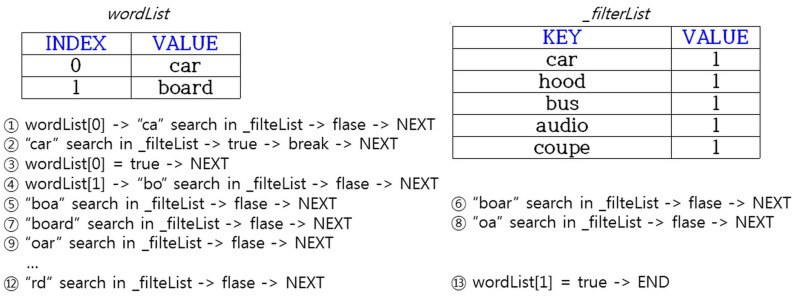
Operation principle of vehicle terminology and profanity filtering function through extraction of audio file text.

When the text of a specific word is detected in the audio file, all Json data are checked and saved, as shown in Table 7. However, the first “fourLetter” and “carLetter” information are set to “false”, and slang and vehicle terms are searched through all detected TEXT information. A total of 2210 terms including Korean and English were used as the profanity filter, and a total of 1700 terms, including Korean and English, were stored in HashMap as the vehicle term filter. The terms added afterward are stored in an Excel file in a certain format and are applied when the file path is delivered as a factor when executed. The principle of extracting the vehicle terms and slang using a filter is shown in Figure 15 below.

If the “car” and “board” from the texts are detected in the JSON file, they are stored in the wordList array using Java’s StringTokenizer class. As the word “car” is stored in the first INDEX, the string is divided in order of “ca”, “car”, and “ar” and searched from the _filterList. The _filterList has a HashMap<String, Integer> structure, and stores words to be filtered in the KEY. The words filtered in the KEY are stored to allow the use of _filterWordList.get(KEY). A KEY-based search is simpler and faster than a VALUE-based search. When searching in _filterList in order of “ca” and “car”, wordList[0] becomes true because it is searched in “car”, and the subsequent search process proceeds. The next step is to divide “board” stored in the second INDEX of wordList in order of “bo”, … “rd” and search the _filterList. However, in this case, wordList[1] becomes false because the final word is not searched.

### 4.3. Video/Voice Process System Manager Service System Implementation

When a user requests a video analysis to the administrator page, the web server executes the Shell Script command with the authority to execute an Ubuntu Linux Shell. In this system, a module that executes the Shell Script command was developed based on the concept of a service router, and each router executes the video and voice processing systems asynchronously. As soon as the video and voice processing system are executed, the administrator can access a specific file of the artificial intelligence server and check the progress of the systems.the sources of execution running on the router can be found in Table 8.

**Figure 16:**
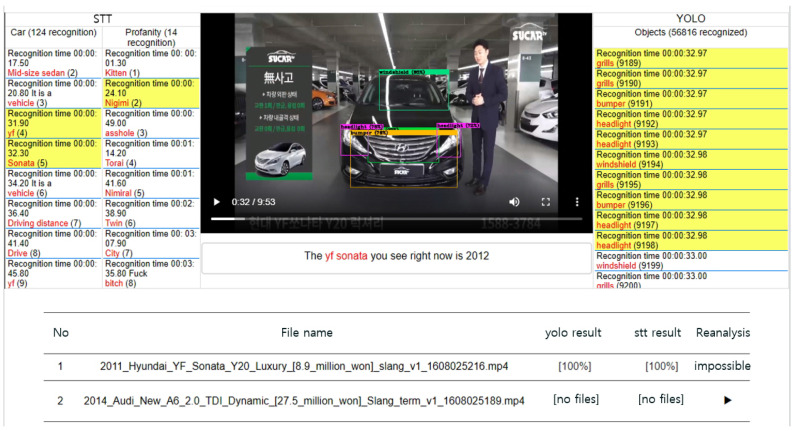
Video analysis screen of administrator service system through video and voice processing systems.

When the executions of both the video and voice processing systems are completed, the progress bar displayed to the user is displayed on the administrator page as 100% along with the completion event, and the file analysis is terminated. Files that have been analyzed can be checked in the list on the web page as shown in the Figure 16, and the analyzed content video and audio subtitles are displayed once the PLAY icon button is clicked. Centering on the video playback, the left layout displays profanity and vehicle-related terms filtered from the audio file, and the right layout displays the vehicle objects recognized in the video. The audio subtitles are displayed at the bottom of the video playback.

## 5. Proposed System Evaluation

### 5.1. Evaluation of System Performance Indicator Goals and Achievements

The system implemented in this study presents the system target performance indicators for commercialization services and an evaluation of the target achievement was requested to the Korea Laboratory Accreditation Scheme (KOLAS) accreditation body. KOLAS is a Korean accreditation organization that performs the task of officially recognizing competence in a specific field by evaluating the inspection, calibration, and testing in Korea in accord with the Framework Act on National Standards and the ISO/IEC Guidelines. The technical performance index goals of the system implemented in this study are shown in Table 9 below.

Ways to measure the system performance goals

- Object recognition accuracy: Upload “vehicle image” and “non-vehicle image” to the server to check whether the object recognition in the image is correct.
- Video stabilization rate: Check whether the artificial intelligence has the correct recognition ability for the contents recorded in the video despite the shakiness during video recording.
- Context awareness rate: Measure the contents of speech recognition in the artificial intelligence server for the recorded voice.
- Profanity slang detection and filtering accuracy: A random user conducts a function test and conducts a questionnaire to measure the performance to detect and filter the content containing profanity and slang from the recorded voice.
- Content upload accuracy: Test the server upload accuracy of edited video.

**Figure 17:**
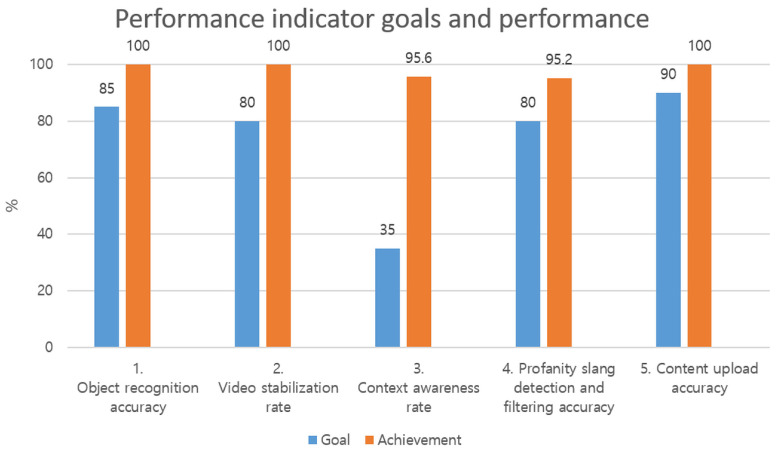
Goals and achievement of the video and voice processing system performance index (KOLAS evaluation result).

The evaluation results of the system performance are shown in Figure 17 below.

As shown in Figure 17 above, the object recognition accuracy was tested in an environment to recognize specific object information, which resulted in a vehicle object recognition rate of 100%, and the vehicle object was recognized at a rate of 100%, even with shaky video. For the context awareness rate according to the speech recognition, text conversion was successful to the extent that there were no problems for the viewer to be aware of the context, and the profanity and slang detection and filtering accuracy functions were also successful with a filtering rate of above 95%. For the uploading of the video content by the user, 100% of the content was uploaded accurately during the test process, which verified the stable performance, and it was finally confirmed that the system was suitably developed for commercialization.

### 5.2. Video Process System Performance Evaluation

#### 5.2.1. Video Process System Performance Evaluation Environment

The success or failure of the stable commercialization service of the system developed in this study depends on the performance of the video process system. This is because the YOLOv3 object recognition algorithm used in the video process system accounts for a large number of computing resources of an artificial intelligence server, and a significant amount of time is spent on the video analysis. Therefore, the performance improvement considered in this study was focused mainly on the video processing system. The system environment for evaluating the performance of the video processing system is as shown in Table 10 below. The utilization rates were compared, and the performance was analyzed for the object recognition speed and graphics card utilization rates of the video process system to which the proposed algorithm was applied with a combination of the YOLOv3 object recognition algorithm, the MSSIM algorithm, and the pure YOLOv3 object recognition algorithm. For the analysis of the object recognition speed, the performance was measured by classifying the image into two methods according to the method stored in the GPU memory when passing the MSSIM algorithm.

#### 5.2.2. Video Process System Speed Evaluation

**Figure 18:**
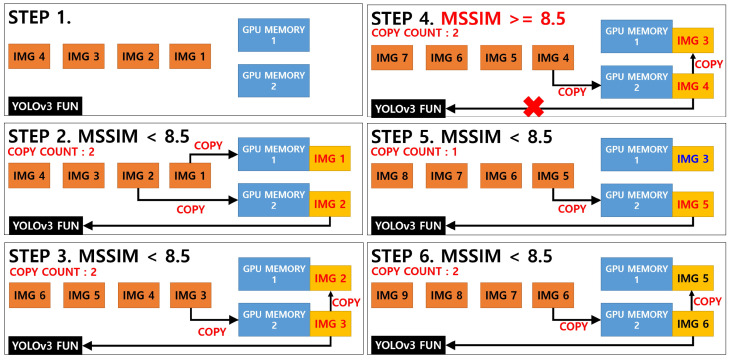
YOLOv3+MSSIM Type 1 image detection system according to MSSIM values; total GPU memory copy count: 9. It is expensive to use the GPU memory.

**Figure 19:**
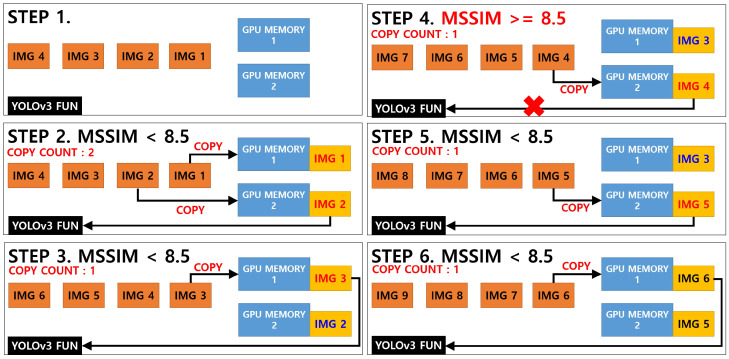
YOLOv3+MSSIM Type 2 image detection system according to MSSIM values; total GPU memory copy count: 6.

The speed evaluation of the video processing system was analyzed by comparing three algorithms. The first algorithm was a pure YOLOv3 object recognition algorithm, and the second and third algorithms were classified according to the way the GPU applies the storage when the image or algorithm developed by combining the YOLOv3 object recognition algorithm and the MSSIM algorithm passes the MSSIM algorithm. The two algorithms were classified into “YOLOv3+MSSIM Type 1” and “YOLOv3+MSSIM Type 2”. The following Figure 18 and Figure 19 describe the YOLOv3 object recognition and MSSIM algorithms.

In Figure 18 and Figure 19 above, images 1 and 2 in “YOLOv3+MSSIM Type 1” are stored in GPU memory, and when the MSSIM value of both images is below 8.5, images 2 and 3 are saved in GPU MEMORY 1 and 2, respectively, during the next step. However, when the MSSIM value of both images is 8.5 or above, image 3 of GPU MEMORY 1 is saved as is, and only the image of GPU MEMORY 2 is replaced, as in steps 4 and 5. As a result, “YOLOv3+MSSIM Type 1” only analyzes the image of GPU MEMORY 2 through the YOLOv3 algorithm. By contrast, in “YOLOv3+MSSIM Type 2” in Figure 19, the number of GPU MEMORY that refers to the YOLOv3 algorithm changes according to the situation. However, the image replacement of GPU MEMORY is not frequently applied because image 3 is continuously fixed to GPU MEMORY 1, as shown in steps 3–5.

Among the videos being commonly uploaded onto online used-vehicle sales platforms, a video script 9 min and 59 s long with many structural changes was used to evaluate the execution speed. More conservative results were expected when there were more structural changes, and the accumulated speed was measured in units of 100 frames while analyzing a total of 35,536 frames. The final cumulative analysis times of the three algorithms were 426.71, 394.32, and 384.71 ms in order of YOLOv3, YOLOv3+MSSIM Type 1, and YOLOv3+MSSIM Type 2. The analysis time efficiencies of approximately 8% in YOLOv3+MSSIM Type 1 and 10% in YOLOv3+MSSIM Type 2 were found compared to the use of pure YOLOv3.

**Figure 20:**
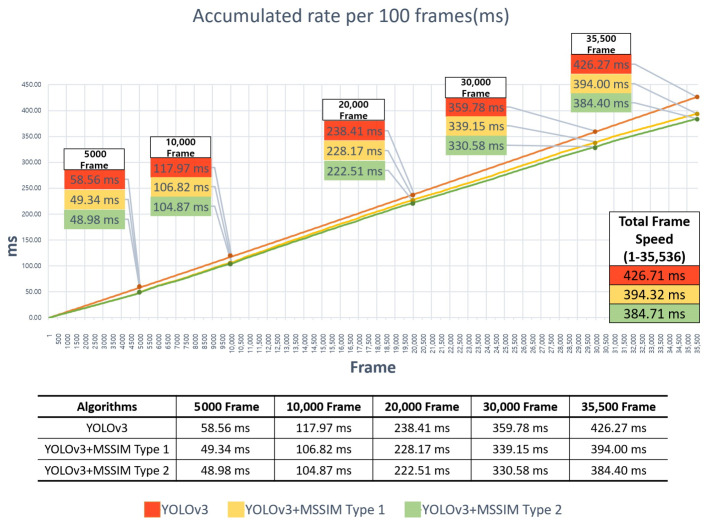
Comparison of object recognition speed per 100 frames of YOLOv3, YOLOv3+MSSIM Type 1, and YOLOv3+MSSIM Type 2.

As shown in the graph in Figure 20 below, it was found that the efficiency of the YOLOv3+MSSIM Type 1 and YOLOv3+MSSIM Type 2 algorithms increase visually after 1,500 frames, and the gap in the graph increases as the number of analyzed frames increases. In a comparison between YOLOv3+MSSIM Type 1 and YOLOv3+MSSIM Type 2, it was found that the efficiency of replacing the resources of the GPU MEMORY is greater than the efficiency of referencing the GPU MEMORY. In other words, because the procedure used for storing and erasing data in the GPU MEMORY significantly affects the computing cost, reducing the data replacement rate of the GPU MEMORY as much as possible can improve the overall computing performance. The following Table 11 compares the number of objects analyzed in 35,536 frames.

In Table 11 above, the number of recognized vehicle objects of the pure YOLOv3 algorithm was 56,816, and the remaining YOLOv3+MSSIM Type 1 and YOLOv3+MSSIM Type 2 algorithms were equally 57,150. In the case of the pure YOLOv3 algorithm, when three objects were recognized in a single frame, two objects were recognized at a small frequency even if the display of the next frame had no structural change from the previous frame. By contrast, for the YOLOv3+MSSIM Type 1 and YOLOv3+MSSIM Type 2 algorithms, when three objects were recognized in the previous frame, the number of cases in which the recognized object was lost was reduced because the recognition result of the previous frame was used when the display of the next frame had no structural change from the previous frame. Such a result does not significantly affect the quality of service owing to the characteristics of the video service but can be a factor proving the improvement of the functional performance of the proposed algorithm. In addition, because the total analysis times of the three algorithms were measured to be less than the video playback time of 599 s, real-time service can be achieved, whereas the analysis time of the YOLOv3+MSSIM Type 2 algorithm can be reduced by 42 s compared to the pure YOLOv3 algorithm, thereby guaranteeing an improvement of the service quality depending on the standby time.

The execution speed and fps were derived by preparing 10 sample videos ranging from 1 to 10 min, which is a commonly used length in online used-vehicle sales platforms, as shown in Table 12 below.

As used-vehicle sales videos generally do not have many structural changes, it was found that the frame rate of the proposed YOLOv3+MSSIM Type 1 and YOLOv3+MSSIM Type 2 algorithms increased, as shown in Table 12 above. Statistical tests were conducted through R-Studio using the above data, and the efficiency of each algorithm was verified by deriving a normal distribution graph and the *t*-test results.

**Figure 21:**
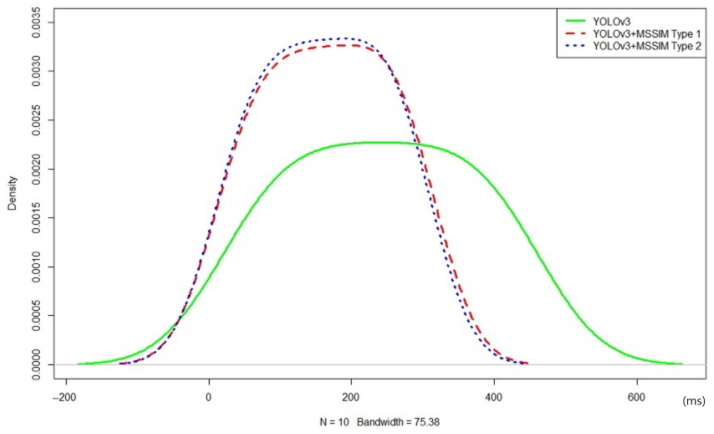
Normal distribution graph of object recognition speed of YOLOv3, YOLOv3+MSSIM Type 1, and YOLOv3+MSSIM Type 2 in videos ranging from 1 to 10 min in length.

YOLOv3 has a wide density based on the time axis x, and YOLOv3+MSSIM Type 1 and YOLOv3+MSSIM Type 2 have a high density around the time axis, as shown in the graph of Figure 21 above. As YOLOv3+MSSIM Type 2 has a slightly denser density than that of YOLOv3+MSSIM Type 1 density, and a larger MAX density, it has been proven to be a more efficient algorithm. The *t*-test results of YOLOv3, YOLOv3+MSSIM Type 1, and YOLOv3+MSSIM Type 2 are as shown in Table 13 below.

As a result of tests 1, 2, and 3 shown in Table 13 above, each *p*-value was less than 0.005 and a reliability of 95% or above was found. In test 1, the difference in the average execution speed was 75.0 ms, which derived more efficient results because the average execution speed of the YOLOv3+MSSIM Type 1 algorithm was faster than that of the YOLOv3 algorithm. Similarly, the difference in average execution speed in test 2 was 78 ms, which resulted in a faster average execution speed of the YOLOv3+MSSIM Type 2 algorithm than the YOLOv3 algorithm. In test 3, the average execution speed of the YOLOv3+MSSIM Type 2 algorithm was 3.59 ms faster than the YOLOv3+MSSIM Type 1 algorithm. Finally, the higher efficiency of the proposed YOLOv3+MSSIM Type 2 algorithm than the conventional YOLOv3 algorithm was verified from the *t*-test results.

An indirect comparison of the average mAP and the frame rate of the conventional AI object recognition algorithm based on the YOLOv3-416 algorithm is shown in Table 14. Although the absolute values were not the same, the YOLOv3-416 algorithm performed the best under a GeForce TITAN X graphics card environment based on the YOLOv3-416 algorithm. Therefore, the proposed YOLOv3+MSSIM Type 2 algorithm can also be expected to perform better than the conventional AI object recognition algorithm under a GeForce TITAN X graphics card environment.

#### 5.2.3. Evaluation of Graphics Card Utilization Rate of Video Process System

To develop the proposed algorithm to a level of commercialization as an automated solution, the operating costs in particular should be reduced. Therefore, the utilization rate of the graphics card, which has not been presented in prior studies on AI object recognition, was verified and evaluated. Such evaluation when applying the video processing system was conducted by comparing and analyzing the three algorithms YOLOv3, YOLOv3+MSSIM Type 1, and YOLOv3+MSSIM Type 2 constructed in this study because no samples were used in prior AI object recognition research studies. The utilization rate of the graphics card was measured in units of 1 s during object recognition for each algorithm, and the efficiency was analyzed using various test techniques.

**Figure 22:**
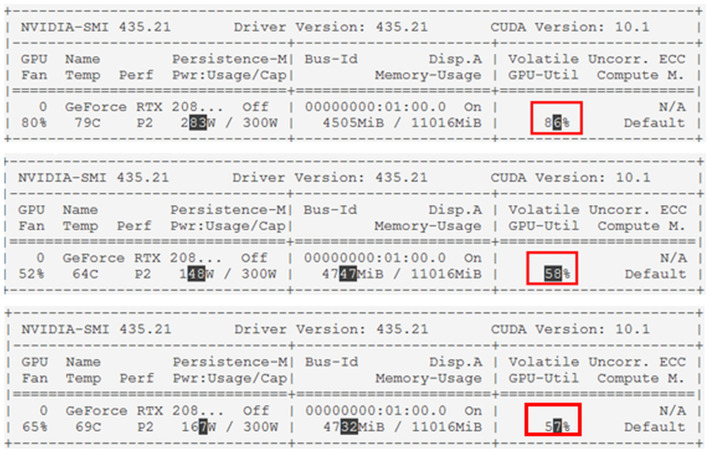
GPU utilization based on Nvidia GeForce RTX 2080Ti using YOLOv3, YOLOv3+MSSIM Type 1, and YOLOv3+MSSIM Type 2.

Figure 22 below shows a captured image of the GPU utilization rate of the YOLOv3, YOLOv3+MSSIM Type 1 and YOLOv3+MSSIM Type 2 algorithms. To analyze the GPU utilization rate of each algorithm, the GPU status was monitored every second while the algorithm was running and stored as a CVS file. Among the stored data, those determined to be meaningless were removed, and the minimum, maximum, and average values of the GPU utilization were derived and analyzed.

**Figure 23:**
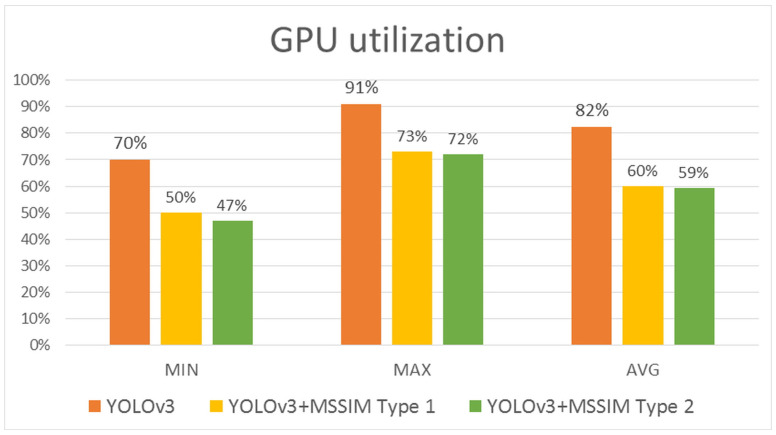
Comparison graph of the minimum, maximum, and average GPU utilization rates of YOLOv3, YOLOv3+MSSIM Type 1, and YOLOv3+MSSIM Type 2.

**Figure 24:**
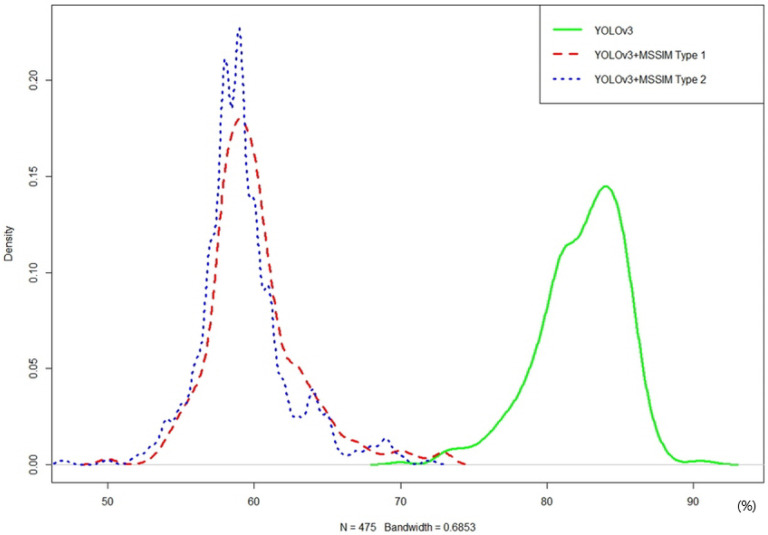
T-test results of YOLOv3, YOLOv3+MSSIM Type 1, and YOLOv3+MSSIM Type 2 for videos ranging from 1 to 10 min in length.

As shown in Figure 23, the pure YOLOv3 algorithm showed a minimum GPU utilization of 70%, a maximum of 91%, and an average of 82%. By contrast, the YOLOv3+MSSIM Type 1 algorithm showed a minimum GPU utilization of 50%, a maximum of 73%, and an average of 60%, demonstrating a higher efficiency than the pure YOLOv3 algorithm. Although the YOLOv3+MSSIM Type 2 algorithm showed a minimum GPU utilization of 47%, a maximum of 72%, and an average of 59%, and showed a higher efficiency than the pure YOLOv3 algorithm, it is difficult to compare the efficiencies of the two algorithms because of their similar results to the YOLOv3+MSSIM Type 1 algorithm. This is because the network computation of the YOLOv3 algorithm consumes more GPU computing resources than the image structural change computations of the MSSIM algorithm. The network computation of the YOLOv3 algorithm was avoided as much as possible and the overall GPU utilization was significantly reduced using the MSSIM algorithm, which has an even number of computations. By contrast, the number of vehicle objects recognized in the image was almost the same, or was increased by keeping significant objects without losing them. The Figure 24 below is a graph of normal distribution derived by R-Studio.

YOLOv3 had a high density around 85% of the GPU utilization axis, and YOLOv3+MSSIM Type 1 and YOLOv3+MSSIM Type 2 had a high density around 60% of the GPU utilization axis as shown in the graph of the Figure 24 above. YOLOv3+MSSIM Type 2 had a slightly higher density around 60% of the GPU utilization axis than that of YOLOv3+MSSIM Type 1, and the MAX density value was also large, thereby proving itself as a more efficient algorithm. The following Table 15 shows the *t*-test result for the CPU utilization rate for each algorithm, for which the *p*-value of each test was less than 0.05, indicating a reliability of 95% or higher.

## 6. Conclusions

In this study, a filter system was developed that can extract vehicle terms and slang words by recognizing vehicle parts as objects and extracting speech from a video for the automated processing of an online vehicle sales platform. The developed key system was divided into video and voice processing systems. The video processing system recognizes the vehicle parts as objects and is configured to be linked with the vehicle sales online platform administrator, whereas the voice processing system uses the STT API to convert speech into text and links the filtered information of the vehicle terms and slang words with the online vehicle sales platform administrator.

**Figure 25:**
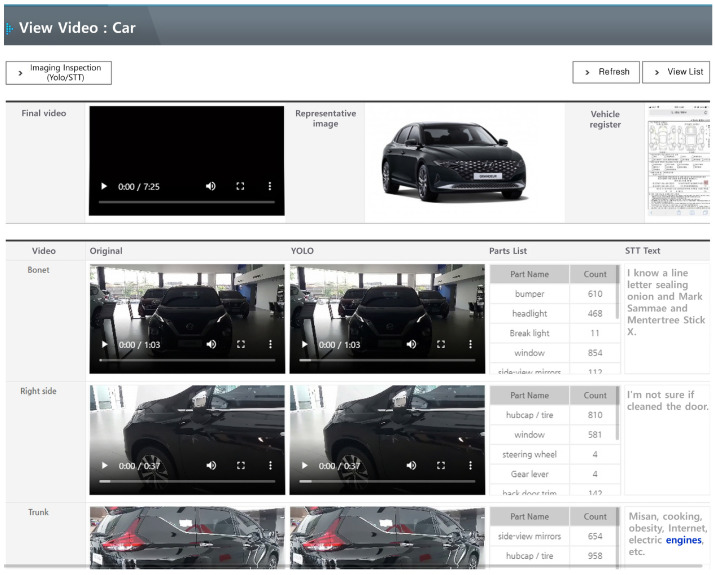
User screen of CIDAUTO vehicle analysis service on online domestic vehicle sales commerce platform.

A module that can call the video and voice processing systems from a web server using a router-based concept was developed for each system to link with the online vehicle sales platform, which allows the administrator to use the function asynchronously by executing commands and sharing file folders. The developed system was applied to an actual commercialized service and launched as a platform product. Figure 25 shows a screenshot of the CIDAUTO product to which the video and voice processing systems were applied.

The video and voice processing systems developed in this study were evaluated by KOLAS, a Korean certification agency, which considers the performance indicators targeted in the initial design and confirmed that the performance targets were achieved. To evaluate the functional performance by comparing the object recognition speed, the number of objects recognized, and the utilization of the graphics card GPU, the video process system was divided into the versions to which the pure YOLOv3 object recognition algorithm, the proposed YOLOv3+MSSIM Type 1 algorithm, and YOLOv3+MSSIM Type 2 algorithm were applied.

As a result of the evaluation, the YOLOv3+MSSIM Type 2 algorithm improved the average execution speed by 78.6 ms, the average frame rate by 40.22 fps, and the average GPU utilization rate by 23.05% compared to the YOLOv3 algorithm, showing that the YOLOv3+MSSIM Type 2 algorithm achieves a better performance under the same resources in the environment and system applied in this study.

In this study, it was found that the current object and speech recognition AI technologies have limitations in application to commercial services. Although the YOLOv3 algorithm can guarantee a better performance than the other object recognition algorithms in terms of the object recognition speed, the use of a graphics card GPU incurs a cost when analyzing only a single video.

It is expected that the present study will present a research direction for institutions and developers aiming to provide AI-based services in the future. This study is also expected to present greater research efficiency and various research directions, particularly for AI object recognition algorithms, by suggesting ways to improve the object recognition speed and reduce the cost of using a GPU without a network modification.

However, the YOLOv3 + MSSIM Type2 algorithm proposed in this study still has a high GPU utilization rate, and inevitably provides limited services when a large amount of work is requested. Therefore, future studies to improve these limitations and provide stable services are needed. Moreover, the pure YOLOv3 algorithm was applied for a comparative evaluation of the YOLOv3+MSSIM Type1 and YOLOv3+MSSIM Type2 algorithms proposed in this study when applied to a video processing system, and the experimental results of previously studied approaches were indirectly compared and analyzed for other AI object recognition algorithms. Therefore, the same test environment needs to be established, and various AI object recognition algorithms need to be used for an accurate comparative analysis in future research.

## Figures and Tables

**Table 1:** Average fps of major AI object recognition algorithms.

Model	Train Dataset	mAP-50(%)	FPS	GPU
YOLOv3-416	COCO Dataset	55.3	35	GeForce TITAN X
YOLOv3-608	COCO Dataset	57.9	20	GeForce TITAN X
SSD300	COCO Dataset	41.2	46	GeForce TITAN X
SSD500	COCO Dataset	46.5	19	GeForce TITAN X
FPN FRCN	COCO Dataset	59.1	6	GeForce TITAN X

**Table 2:** Definition of design requirements according to the video processing system function.

Item	Contents
Object recognition Artificial Intelligence	System administrator to learn the videos on the vehicle and vehicle parts
	Create vehicle and vehicle parts object recognition model in video
	Video analysis by system request
Video analysis results	Save object recognition analysis result log
	Transmit video analysis results to the system (JSON)
Others	Management of uploaded video and learning data
	Save uploaded video and learning data by directory

**Table 3:** Definition of design requirements according to voice processing system function.

Item	Contents
Supported language	Support Korean language recognition
	Support various foreign languages (including Southeast Asian countries)
Voice recognition result	Filter vehicle description and profanity
	Function to add vehicle description and profanity after system development
	Save voice filtering result log
	Transmit voice filtering results to the system (JSON)
Others	Utilizing STT modules from stable service providers for continuous service

**Table 4:** Vehicle object items defined to recognize YOLOv3-based vehicle parts.

Item	Content and Object No.
Vehicle external parts	(0) headlight, (1) bumper, (2) hood, (3) grills, (4) window, (5) engine, (6) Trunk, (7) Break light, (8) hubcap/tire, (9) side-view mirrors, (10) windshield, (11) front door trim, (12) back door trim, (13) steering wheel
Vehicle internal parts	(14) center fascia, (15) glove box, (16) instrument board, (17) gear lever, (18) air conditioner, (19) rear seats

**Table 5:** JSON format of vehicle part recognition results for vehicle part and image information.

Format Variable Name	Format Definition
frame_id	Current frame number
filename	File name of input video
total_frame	Total number of frames in the input video
fps	Frame rate of input video
time	Video playback time of the current frame
objects	An array was defined to display the recognized vehicle part object information (class_id, name), the position and size in the image (realativ_coordinates), and the confidence regarding the recognized object.

**Table 6:** Cause analysis of the analyzed frame sync error and sync error fixes.

Title 1	Title 2
Cause of sync error	The first and last frames in the original file are dropped
Sync error fixes	(1) Copied and encoded the first frame dropped from the original file (2) Copied and encoded the frame dropped from the last frame. (3) Synchronized frame of the original and the copy with the recognized object

**Table 7:** JSON format of speech recognition results for extracting profanity and related words.

Format Variable Name	Format Definition
index	Index of word extracted from audio file
fourLetter	Variable determining whether the currently extracted text is slang
startTime	The moment when the speech of the extracted text first started
endTime	The moment at which the speech of the extracted text ended
word	Text extracted from audio files
carLetter	Variable that determines whether the currently extracted text has a vehicle-related terminology

**Table 8:** Router execution source of video and voice processing systems.

Item	Router Execution Source
Video Process System	$cmd = “./darknet detector demo data/obj.data data/yolo-obj.cfg backup/yolo-obj_final.weights \“upload/{$file}.mp4\” -out_filename \“upload/{$file}_yolo.mp4\””; $pid = backgroundExec($cmd);
Voice Process System	$cmd. = “java -jar speech-+-cloud.jar wordoffsets \“../yolo/upload/{$file}.mp4\” {$lencode}”; $pid = backgroundExec($cmd);

**Table 9:** Performance indicators and target achievement values for a performance evaluation of the video and voice processing systems (KOLAS accredited target achievement).

Evaluation Item (Main Performance Spec)	Unit	Proportion (%)	Development Target
1. Object recognition accuracy	%	25	85% or above
2. Video stabilization rate	%	15	80% or above
3. Context awareness rate	%	25	35% or above
4. Profanity slang detection and filtering accuracy	%	15	80% or above
5. Content upload accuracy	%	20	90% or above

**Table 10:** Video and voice processing system construction environment.

Item	Specification	Remarks
CPU	Intel Core 9 Gen i7-9700k (4.90 GHz)	-
Motherboard	ASUS PRIME Z390-A STCOM (Intel Z390/ATX)	-
RAM	DDR4 64 GB (DDR4 16GB × 4)	Samsung DDR4 16 GB PC4-21300
OS	Ubuntu Desktop	version: 18.0.4 LTS
LAN	port 1 (internal)—10/100 Mbps port 2 (external)—10/100 Mbps	-
Storage	SSD: 512 GB/HDD: TB (2 TB × 2)	Total: 4.5 TB
GPU	GPU 1—GeForce RTX 2080 Ti 11 GB	-
Power	1000W (+12 V Single Rail)	Micronics Performance II HV 1000 W Bronze

**Table 11:** Comparison table of total analysis time, average fps, and total number of recognized objects for YOLOv3, YOLOv3+MSSIM Type 1, and YOLOv3+MSSIM Type 2.

Item	Total Speed (35,536 Frame)	Average FPS	Total Object (35,536 Frame)
YOLOv3	426.710524 ms	83.29 FPS	56,816
YOLOv3+MSSIM Type 1	394.329463 ms	90.11 FPS	57,150
YOLOv3+MSSIM Type 2	384.718328 ms	92.37 FPS	57,150

**Table 12:** Total analysis time and average fps of YOLOv3, YOLOv3+MSSIM Type 1, and YOLOv3+MSSIM Type 2 in videos ranging from 1 to 10 min in length.

Item	YOLOv3		YOLOv3+MSSIM Type 1		YOLOv3+MSSIM Type 2	
	Analysis Time	Average FPS	Analysis Time	Average FPS	Analysis Time	Average FPS
1 min	43.35 ms	82.95 FPS	30.83 ms	116.63 FPS	30.06 ms	119.64 FPS
2 min	86.66 ms	83.00 FPS	55.35 ms	129.96 FPS	54.37 ms	132.29 FPS
3 min	131.39 ms	82.11 FPS	91.61 ms	117.76 FPS	89.26 ms	120.86 FPS
4 min	174.82 ms	82.26 FPS	118.79 ms	121.06 FPS	115.86 ms	124.12 FPS
5 min	218.91 ms	82.15 FPS	154.14 ms	116.66 FPS	151.00 ms	119.09 FPS
6 min	262.88 ms	82.07 FPS	178.13 ms	121.11 FPS	174.50 ms	123.64 FPS
7 min	306.38 ms	82.17 FPS	215.24 ms	116.96 FPS	209.89 ms	119.95 FPS
8 min	351.28 ms	81.91 FPS	245.38 ms	117.26 FPS	240.48 ms	119.64 FPS
9 min	394.16 ms	82.11 FPS	269.05 ms	120.29 FPS	263.54 ms	122.80 FPS
10 min	437.08 ms	82.28 FPS	298.31 ms	120.55 FPS	291.93 ms	123.18 FPS

**Table 13:** T-test results of YOLOv3, YOLOv3+MSSIM Type 1, and YOLOv3+MSSIM Type 2 in videos ranging from 1 to 10 min in length.

Test No.	Compared Algorithm	Average Execution Speed	Average fps	Average Execution Speed Difference	T-Value	*p*-Value
1	YOLOv3	240.69 ms	82.30 FPS	75.0 ms	5.7331	0.00028
	YOLOv3+MSSIM Type 1	165.68 ms	119.82 FPS			
2	YOLOv3	174.82 ms	82.30 FPS	78.6 ms	5.7484	0.00027
	YOLOv3+MSSIM Type 2	162.09 ms	122.52 FPS			
3	YOLOv3+MSSIM Type 1	165.68 ms	119.82 FPS	3.59 ms	5.9151	0.00022
	YOLOv3+MSSIM Type 2	162.09 ms	122.52 FPS			

**Table 14:** Comparison of object recognition algorithm performance.

Model	Train Dataset	mAP-50(%)	FPS	GPU
YOLOv3+MSSIM Type 1	Our	94.23	120	GeForce RTX 2080 TI
YOLOv3+MSSIM Type 2	Our	94.23	125	GeForce RTX 2080 TI
YOLOv3-416	Our	94.23	82	GeForce RTX 2080 TI
YOLOv3-416 [21]	COCO Dataset	55.3	35	GeForce TITAN X
YOLOv3-608 [21]	COCO Dataset	57.9	20	GeForce TITAN X
SSD300 [42]	COCO Dataset	41.2	46	GeForce TITAN X
SSD500 [42]	COCO Dataset	46.5	19	GeForce TITAN X
SSD321 [43]	COCO Dataset	45.4	16	GeForce TITAN X
DSSD321 [43]	COCO Dataset	46.1	12	GeForce TITAN X
FPN FRCN [1]	COCO Dataset	59.1	6	GeForce TITAN X

**Table 15:** T-test results of GPU utilization for YOLOv3, YOLOv3+MSSIM Type 1, and YOLOv3+MSSIM Type 2.

Test No.	Compared Algorithm	Average GPU Utilization	Average GPU Utilization Differences	T-Value	*p*-Value
1	YOLOv3	82.34%	22.29%	109.09	*p* < 0.0001
	YOLOv3+MSSIM Type 1	60.05%			
2	YOLOv3	82.34%	23.05%	116.28	*p* < 0.0001
	YOLOv3+MSSIM Type 2	59.29%			
3	YOLOv3+MSSIM Type 1	60.05%	0.76%	3.6293	0.0002
	YOLOv3+MSSIM Type 2	59.29%			
